# Supplementary material for: Diffusion- and Chemometric-Based Separation of Complex Electrochemical Signals That Originated from Multiple Redox-Active Molecules
Source: Polymers (Basel). 2022 Feb 13;14(4):717. doi: 10.3390/polym14040717 (PMC8875081; doi:10.3390/polym14040717)
Supplement: Supplementary file 1 [file polymers-14-00717-s001.zip › polymers-1545287-supplementary.pdf]

Supporting Information

Diffusion- and Chemometric-Based Separation of Complex Electrochemical Signals that Originated from Multiple Redox-Active Molecules

Stav Biton Hayun, Rajendra P. Shukla and Hadar Ben-Yoav *

Nanobioelectronics Laboratory (NBEL), Department of Biomedical Engineering, Ilse Katz Institute of
Nanoscale Science and Technology, Zlotowski Center for Neuroscience, Ben-Gurion University of the Negev, Beer-Sheva 8410501, Israel;
stavbi@post.bgu.ac.il (S.B.H.); rajendra@post.bgu.ac.il (R.P.S.)
*   Correspondence: benyoav@bgu.ac.il; Tel.: +972-08-6479717

Table S1. Effective diffusion coefficients calculated for ascorbic acid (AA), clozapine (CLZ), ferricyanide (Ferri), L-homocysteine (HCy), and uric acid (UA) for different chitosan film thicknesses.

	Redox-active molecule	Thickness of the chitosan film (nm)					
		Bare	33.4	40.9	54.7	72	84.1
Effective diffusion coefficient [$cm^2 s^{-1}$]	AA	$4.53\times10^{-9} \pm 3.43\times10^{-13}$	$4.87\times10^{-9} \pm 1.19\times10^{-12}$	$5.34\times10^{-9} \pm 4.12\times10^{-13}$	$4.05\times10^{-9} \pm 1.21\times10^{-12}$	$7.53\times10^{-9} \pm 3.13\times10^{-12}$	$3.56\times10^{-9} \pm 1.59\times10^{-13}$
	CLZ	$7.45\times10^{-7} \pm 4.22\times10^{-10}$	$3.26\times10^{-7} \pm 9.99\times10^{-11}$	$4.61\times10^{-8} \pm 4.46\times10^{-12}$	$1.40\times10^{-7} \pm 4.96\times10^{-11}$	$1.03\times10^{-7} \pm 7.11\times10^{-11}$	$1.05\times10^{-7} \pm 1.96\times10^{-11}$
	Ferri	$6.85\times10^{-8} \pm 6.49\times10^{-9}$	$1.27\times10^{-7} \pm 4.43\times10^{-8}$	$8.38\times10^{-8} \pm 1.17\times10^{-8}$	$5.80\times10^{-8} \pm 2.36\times10^{-8}$	$4.52\times10^{-8} \pm 1.68\times10^{-8}$	$5.93\times10^{-8} \pm 1.65\times10^{-8}$
	HCy	$3.01\times10^{-9} \pm 8.04\times10^{-13}$	$1.64\times10^{-9} \pm 9.09\times10^{-13}$	$1.50\times10^{-9} \pm 4.21\times10^{-13}$	$1.02\times10^{-9} \pm 7.79\times10^{-13}$	$5.63\times10^{-10} \pm 4.07\times10^{-13}$	$7.87\times10^{-10} \pm 4.87\times10^{-13}$
	UA	$2.08\times10^{-9} \pm 2.23\times10^{-13}$	$1.78\times10^{-9} \pm 3.78\times10^{-13}$	$3.32\times10^{-9} \pm 2.65\times10^{-13}$	$2.03\times10^{-9} \pm 4.55\times10^{-13}$	$3.30\times10^{-9} \pm 3.56\times10^{-13}$	$2.28\times10^{-9} \pm 3.58\times10^{-13}$

Table S2. The composition of solutions used for the chemometric PLSR model training.

Solution number	Redox-active molecule concentration (μM)			
	CLZ	UA	HCy	AA
1	3.75	260	500	500
2	1	260	500	1000
3	3.75	140	300	1000
4	3.75	380	400	500
5	3.75	140	150	500
6	2.5	260	400	1000
7	1	500	500	1000
8	1	260	300	1000
9	1	500	400	1000
10	5	260	400	200
11	3.75	140	300	200
12	5	260	400	500
13	3.75	140	400	200
14	1	140	300	200
15	5	380	500	500
16	1	260	300	200
17	3.75	260	150	1000
18	5	260	300	0
19	3.75	140	300	0
20	2.5	140	400	500
21	1	260	300	500
22	3.75	380	300	1000

23	5	140	400	200
24	3.75	500	400	200
25	2.5	380	300	500
26	1	500	400	500
27	1	140	500	500
28	3.75	500	150	500
29	2.5	260	150	500
30	2.5	380	300	200
31	1	380	150	500
32	5	500	150	1000
33	3.75	380	400	1000
34	1	500	150	0
35	3.75	260	500	0
36	2.5	260	400	0
37	3.75	500	500	200
38	3.75	500	150	0
39	2.5	140	150	1000
40	1	140	500	0
41	3.75	380	500	0
42	2.5	380	500	1000
43	2.5	260	500	200
44	1	140	400	0
45	2.5	140	150	1000
46	2.5	380	500	0
47	2.5	260	150	200
48	1	380	150	200
49	5	500	500	200

50	2.5	500	500	200
51	5	500	300	500
52	5	380	400	1000
53	2.5	500	300	500
54	1	380	400	0
55	5	140	150	0
56	5	140	500	1000
57	2.5	500	400	0
58	5	260	150	0
59	2.5	500	300	0
60	5	500	300	1000
61	5	380	150	200
62	1	380	150	200
63	5	140	500	500
64	5	380	300	0

Table S3. The composition of solutions used for testing the trained chemometric PLSR model.

Solution number	Molecule concentration (µM)			
	CLZ	UA	HCy	AA
1	3.75	260	500	500
2	5	500	150	1000
3	3.75	500	400	200
4	1	140	500	1000
5	2.5	380	300	500
6	5	380	300	0
7	1	260	300	0
8	1	380	150	200
9	2.5	260	500	200
10	2.5	140	400	500
11	5	380	400	1000
12	5	260	300	0
13	3.75	260	300	0
14	2.5	380	300	200
15	3.75	380	500	0
16	5	260	150	1000
17	3.75	380	400	500
18	2.5	380	500	0
19	1	380	400	0
20	3.75	380	150	1000
21	5	380	150	0
22	5	260	150	500

23	3.75	140	150	200
24	5	260	400	500
25	5	500	150	1000
26	4.25	200	350	400
27	4.6	430	215	0
28	1.5	300	460	700
29	3.5	325	270	0
30	2	400	420	900
31	4.8	175	245	0

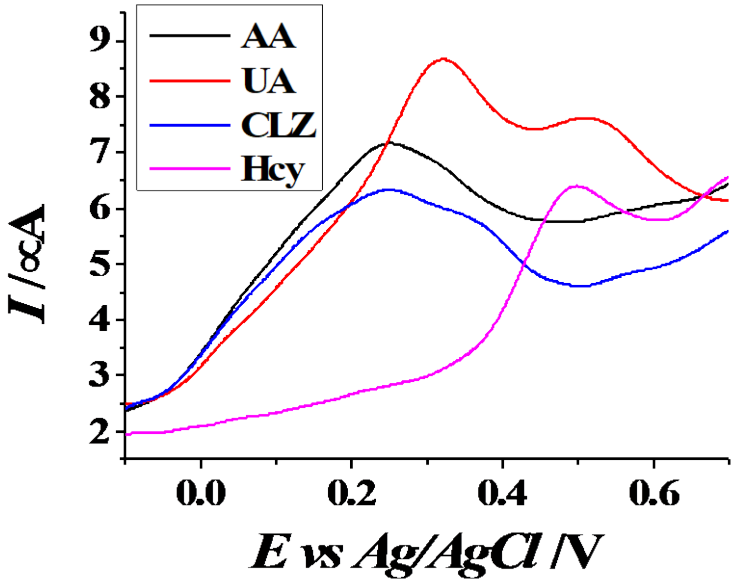

Figure S1. The electrochemical signatures measured from the same concentration of ascorbic acid (AA; black), uric acid (UA; red), clozapine (CLZ; blue), and L-homocysteine (Hcy; magenta) by using a gold bare electrode.

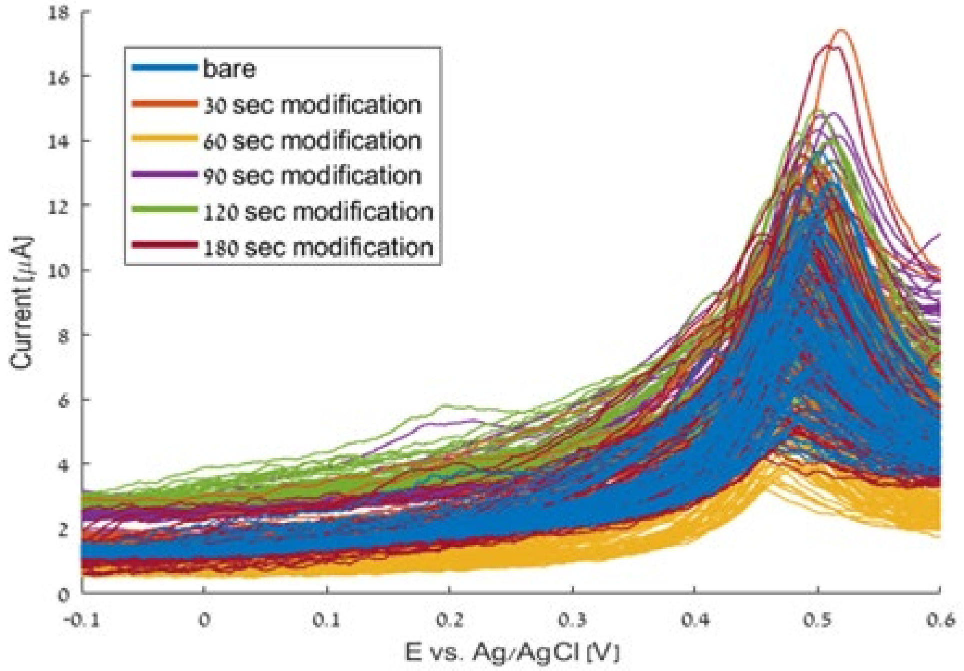

Figure S2. Example of the differential pulse voltammograms recorded from the different mixture solutions by using a bare electrode (blue) and electrodes modified with 30s- (orange), 60s- (yellow), 90s- (purple), 120s- (green), and 180s- (brown) chitosan electrodeposited electrodes.

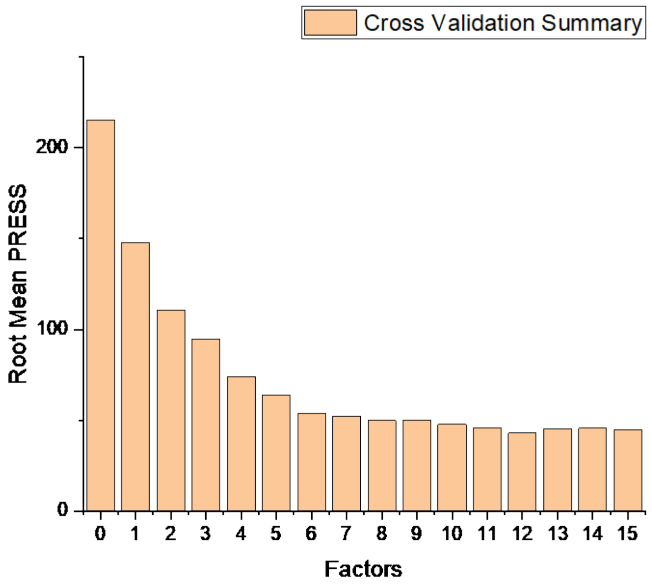

Figure S3. Root mean predicted residual error sum of squares (PRESS) calculated by a leave-one-out cross-validation.

Table S4. Explained X and Y variance.

Number of Factors	Variance Explained for X Effects (%)	Cumulative X Variance (%)	Variance Explained for Y Responses (%)	Cumulative Y Variance (%)
1	68.62809	68.62809	54.22085	54.22085
2	11.45509	80.08318	22.44814	76.66899
3	5.21964	85.30282	7.27516	83.94415
4	2.16823	87.47105	8.41452	92.35867
5	3.20797	90.67901	2.40783	94.7665
6	1.46711	92.14612	1.72614	96.49265
7	0.80359	92.94972	0.79287	97.28552
8	0.90078	93.85049	0.48951	97.77503
9	1.49947	95.34997	0.21606	97.99109
10	0.55315	95.90311	0.44503	98.43613
11	0.89668	96.79979	0.16075	98.59688
12	0.29571	97.0955	0.21004	98.80691
